# Systematic review and meta-analysis of the relationship between sleep disorders and suicidal behaviour in patients with depression

**DOI:** 10.1186/s12888-019-2302-5

**Published:** 2019-10-17

**Authors:** Xiaofen Wang, Sixiang Cheng, Huilan Xu

**Affiliations:** Department of Social Medicine and Health Management, Xiangya School of Public Health, Central South University, NO.238 Shang Ma Yuan Ling Road, Kaifu District, Changsha, 410078 Hunan Province China

**Keywords:** Sleep disorder, Suicidal behaviour, Depression, Systematic review, Meta-analysis

## Abstract

**Background:**

The potential link between sleep disorders and suicidal behaviour has been the subject of several reviews. We performed this meta-analysis to estimate the overall association between sleep disorders and suicidal behaviour and to identify a more specific relationship in patients with depression.

**Methods:**

A systematic search strategy was developed across the electronic databases PubMed, EMBASE and the Cochrane Library from inception to January 1, 2019 for studies that reported a relationship between sleep disorders and suicidal behaviour in depressed patients. The odds ratio (OR) and corresponding 95% confidence interval (CI) were used to measure the outcomes. Heterogeneity was evaluated by Cochran’s Q test and the I^2^ statistic. The Newcastle-Ottawa Scale (NOS) was adopted to evaluate the methodological quality of each of the included studies, and the Grading of Recommendations Assessment, Development and Evaluation (GRADE) approach was used to assess the quality of the evidence. We calculated the overall association between sleep disorders and suicidal behaviour and estimated more specific categories, including insomnia, nightmares, hypersomnia, suicidal ideation, suicide attempt, and completed suicide.

**Results:**

A total of 18 studies were included in this study. Overall, sleep disorders were closely related to suicidal behaviour in patients with depression (OR = 2.45 95% CI: 1.33 4.52). The relatively increased risks of sleep disorders with suicidal ideation, suicide attempt and completed suicide ranged from 1.24 (95% CI: 1.00 1.53) to 2.41 (95% CI: 1.45 4.02). Nightmares were found to be highly correlated with the risk of suicidal behaviour (OR = 4.47 95% CI: 2.00 9.97), followed by insomnia (OR = 2.29 95% CI: 1.69 3.10). The certainty of the evidence was rated as very low for the overall outcome and the major depression subgroup and was rated as low for the depression subgroup.

**Conclusions:**

This meta-analysis supports the finding that sleep disorders, particularly nightmares and insomnia, increase the risk of suicidal behaviour in depressed patients. Considering that all included studies were observational, the quality of the evidence is rated as very low. More well-designed studies are needed to confirm our findings and to better explain the mechanisms by which sleep disorders aggravate suicidal behaviour in depressed patients.

## Background

Suicide is a major public health problem worldwide. Globally, it is estimated that more than 800,000 people died of suicide in 2012, and more than 1 million people died of suicide in 2016 [1, 2]. In the United States, suicide is the second most common cause of death in people aged 15~34 years old. Similarly, in China, suicide is the leading cause of death in people in the same age group [3]. Millions of individuals are bereaved by suicide every year, and suicide prevention has overwhelming significance for global public health. The Institute of Medicine (IOM) has made suicide prevention a top priority and called for measures to explore evidence-based risk factors to reduce national suicide rates [4]. To date, many risk factors have been described, including unchangeable and potentially changeable factors. Unchangeable factors include age, gender, and severe physical illness, whereas changeable factors include previous suicide attempts, harmful use of alcohol and insomnia symptoms [5–7]. Among these factors, global or partial insomnia is an important risk factor for suicidal behaviour, but it has generally been overlooked in previous reviews of suicide risk factors and suicide prevention [8, 9].

Depressive disorders are the most prevalent mental illness and a well-established risk factor for suicidality [10]. According to the World Health Organization (WHO), 350 million people worldwide suffer from depression, and major depression is expected to be a major cause of disability by 2030 [11, 12]. Additionally, the suicide rate in depressed patients is reported to be 22 to 36 times higher than that in the general population [13]. The incidence of suicidal ideation in patients with major depression is approximately 11% ~ 63% [14], among which 15% have recurrently attempted suicide [15]. People with depression present many physical symptoms in addition to the diagnostic criteria, such as somatic pain, gastrointestinal symptoms and sleep disorders [16]. Sleep disorders, particularly insomnia, are reported to be a core symptom of depression and double the risk of developing depression [17]. Severe insomnia in the diagnosis of depression has been confirmed as one of the few clinical predictors of suicide in the first year of follow-up [18].

Numerous studies have shown that both depression and sleep disorders are independent risk factors for suicidal behaviour. Do sleep disorders increase the risk of suicidal behaviour in depressed patients? Epidemiological evidence remains controversial. Some studies have shown that suicide in depressed patients is mainly caused by the clinical symptoms of the disease itself and have proposed a genetic hypothesis based on animal experiments [19, 20]. Through a national survey, Fang et al. found that both late insomnia and hypersomnia were strongly associated with suicidal ideation in the first episode of major depression in Chinese patients [21]. However, Chellappa et al. confirmed that only insomnia was associated with suicidal intention after controlling for age and gender [18]. Conversely, Li et al. reported that nightmares had a significant association with suicidal behaviour, and the link between insomnia and suicide disappeared in multivariate analysis [22]. According to Gallagher et al., sleep disorders in depressed patients were significantly associated with a higher risk of completed suicide after adjusting for age and gender but not after fully adjusting for other variables [23].

Considering the high suicide rate and high prevalence of sleep disorders in patients with depression, we conducted this systematic review and meta-analysis to address the disagreement between several previous research articles and to provide valuable information for clinical practice by exploring the risk difference between various sleep disorders and suicidal behaviours.

## Methods

Based on the Systematic and Meta-analytical Preferred Reporting Program (PRISMA) [24], a prospective protocol involving objectives, search strategies, inclusion and exclusion criteria, outcomes and methods of system analysis was developed before our implementation of this study. The detailed PRISMA checklist can be found in Additional file 1: Table S1.

### Literature search strategy

A systematic search strategy with no language, geographical, or publication-type restrictions was developed in January 2019. Our search consisted of two steps. First, we searched the electronic databases PubMed, EMBASE and the Cochrane Library from inception to January 1, 2019. We used the following Medical Subject Headings (MeSH) terms and their combinations of free words to search: *sleep disorders/sleep arousal disorders/sleep deprivation//sleep wake disorders/sleep initiation and maintenance disorders, depressive disorder/depression/mental disorders*, and *suicidal behaviour/suicide ideation/suicide attempt /suicide.* Second, we implemented a manual search based on the reference lists of the articles included in the first step and contacted the authors via email to obtain unpublished data. We supplemented the results of electronic searches with manually searched comments, reviews, and conference abstracts. The detailed search strategy can be found in Additional file 1: Table S2. The entire search was independently performed by two reviewers (XFW & SXC).

### Study selection and data extraction

We included all observational studies that reported an association between sleep disorders and suicidal behaviour in patients with depression. “Depression” in this study referred to the definition of the diagnosis of depression (including major depression) according to internationally recognized diagnostic tools, excluding patients with depressive symptoms but diagnosed with mental illnesses other than depression, such as bipolar disorder. If the original study clarified that the patient’s diagnosis was depression, our default assumption was that they had excluded a disorder such as bipolar disorder that needed to be excluded in the diagnosis of depression. According to the inclusion criteria of the original studies, if the author reported major depressive disorder (MDD) or indicated that the subject’s depression was severe, we assigned this study to the “major depression” group. If the author only mentioned depression and did not report the severity of the patient’s depression, we assigned the study to the “depression” group. The classification of sleep disorders was broad and included insomnia, hypersomnia, nightmares, and various sleep problems defined by the authors in their original studies, such as low sleep quality, short sleep duration, and low habitual sleep efficiency. Suicidal behaviour in this meta-analysis included suicidal ideation, suicide attempt and completed suicide. Studies were excluded if they (a) studied an irrelevant topic, used an unavailable population or had incomplete data, (b) were reviews, conference abstracts, editorial letters, qualitative research or animal model papers, or (c) were duplicated reports. If there were duplicate studies, the most recent or most complete version was selected. If multiple languages were used to describe and publish the same data, the English version was selected. We extracted data on the first author, year of publication, country of origin, study design, participant numbers and characteristics, exposure and outcomes and their measurement tools. The data from all included studies were extracted and reviewed independently by XFW and SXC. Any dispute or disagreement between the two reviewers that could not be agreed upon was arbitrated by a third reviewer (HLX).

### Assessment of risk of bias and grading the evidence

The methodological quality of all included studies was independently assessed using the modified version of the Newcastle-Ottawa Scale (NOS) [25]. Potential risk of bias was identified by five items, including representativeness of the subjects, diagnosis of depression, adjustment for confounders, ascertainment of outcomes and reporting loss to follow-up. The risk of each item was identified as “low risk”, “high risk” or “unclear risk”. Low risk was recorded as “1 point”, and the other two types of risk were recorded as “0 points”. A study with a total score of 3 points or above indicated a lower risk of bias.

Confidence in the estimate for the outcome was determined by the Grading of Recommendations Assessment, Development and Evaluation (GRADE) approach [26]. This assessment was performed by GRADEpro software (version 3.2, Evidence Prime, Hamilton, ON, Canada, 2015) [27]. The rating criteria were based on risk of bias, inconsistency, indirectness, imprecision, publication bias and other considerations. Because all included studies in this meta-analysis were observational studies, the quality of the evidence was initially defined as low. However, the rating could be upgraded to high (a) if the magnitude of the treatment effect was large (relative risk (RR) > 2 or RR <  0.5) or very large (RR > 5 or RR <  0.2), (b) if there was evidence of a dose-response relation or (c) if all plausible biases could decrease the magnitude of an apparent treatment effect. In addition, according to the handbook of GRADE, because inconsistency in this study could be explained by differences in populations (depression *vs*. major depression), we analysed, presented and graded the quality of the evidence by stratifying according to this variable.

### Outcomes and statistical analysis

The result of interest was the relationship between sleep disorders and suicidal behaviour. Sleep disorders included insomnia, nightmares, and hypersomnia; no specific sleep disorder was classified as “other”. Suicidal behaviour included suicidal ideation, suicide attempt and completed suicide. Studies that did not specifically address suicidal behaviour were also classified as “other”. The association between sleep disorders and suicidal behaviour was expressed as the odds ratio (OR). We obtained the ORs and corresponding 95% confidence intervals (CI) from most of the included studies. If those values were not directly available, we calculated the OR and 95% CI with the coefficient estimates and standard errors mentioned in the text.

The statistical analysis was conducted using Stata 12.0 (College Station, TX: StataCorp LP) and RevMan 5.3 (Cochrane Collaboration, Copenhagen, Denmark). Outcomes were assessed by pooled logOR among all included studies, and the corresponding 95% CIs were calculated by a fixed effects model or a random effects model. Statistical heterogeneity was defined by Cochran’s Q test with a *P* value < 0.1 or an I^2^ value > 50%, and a random effects model was used for subsequent analysis. Conversely, when the *P* value was > 0.1 or the I^2^ value was < 50%, a fixed effects model was used. To explore potential sources of heterogeneity, meta-regression and subgroup analyses were implemented through several categories: year of publication (1997 ~ 2010 *vs*. 2010 ~ 2019), study design (retrospective *vs*. prospective *vs*. cross-sectional), sample size (< 1000 *vs*. > 1000), age of subjects (mean < 40 *vs*. mean > 40), diagnostic outcome (major depression *vs*. depression), diagnostic criteria (ICD-10 *vs*. DSM-III-R *vs*. DSM-IV *vs*. CES-D), sleep disorder type (insomnia *vs.* nightmares *vs.* hypersomnia *vs.* other), and suicidal behaviour type (suicidal ideation *vs.* suicide attempt *vs.* completed suicide *vs.* other). Potential publication bias was detected by testing the asymmetry of the funnel plot and performing an Egger’s linear regression test. Sensitivity analysis was determined by excluding studies with a relatively high risk of bias and comparing the results to previous effect values. *P* <  0.05 was considered statistically significant in all analyses.

## Results

### Study identification and selection

**Fig. 1:**
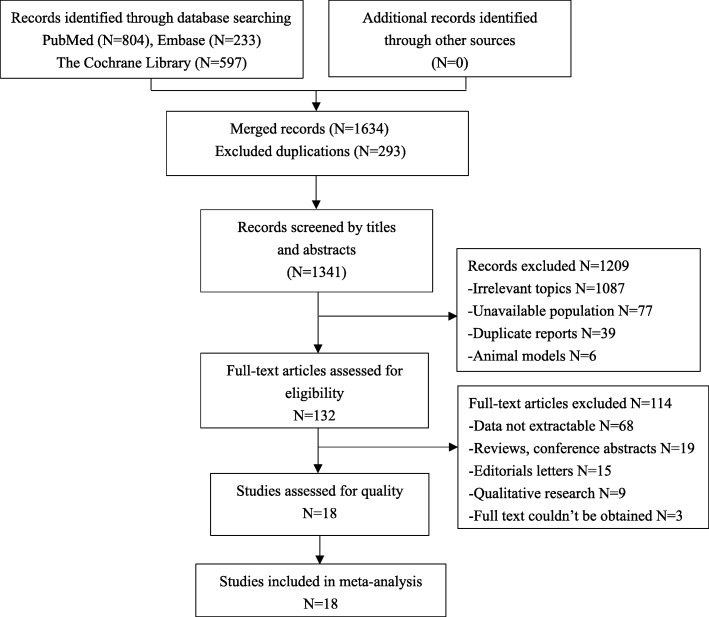
Flow diagram of included/excluded studies. A total of 1634 documents were found in the initial search. After removing duplicates, reading titles, abstracts and full texts, and evaluating the quality of the articles, 18 eligible studies were included and analysed

A total of 1634 studies were identified through a preliminary database search, and 293 duplicates were excluded from Endnote X8 (Thomson Reuters, MI). By browsing the titles and abstracts, 132 studies were available and entered full-text screening. After reading these documents carefully, 114 studies were excluded (68 had data that were not extractable, 19 were reviews or conference abstracts, 15 were editorial letters, 9 were qualitive research studies, and 3 full texts could not be obtained). Finally, a total of 18 eligible studies were included in this meta-analysis. The detailed research identification and selection are shown in Fig. 1.

### Study characteristics

The 18 studies were published between 1997 and 2019, covering a total of 198,893 participants whose mean ages in each study ranged from 12.5 (SD =3.1) to 48.1 years (SD =15.6). There were 6 retrospective studies, 6 prospective studies and 6 cross-sectional studies. The sample sizes in each study varied from 41 to 163,512, and all patients were formally diagnosed with depression or major depression. Regarding the diagnostic criteria, the Diagnostic and Statistical Manual of Mental Disorders (DSM) was used in most studies (15), including the DSM-III-R (4) and the DSM-IV (11); the International Classification of Diseases, Tenth Revision (ICD-10) was adopted in two studies; and the Center for Epidemiology Scale for Depression (CES-D) was used in one study. The sleep disorders we included were hypersomnia, insomnia, sleep quality, etc. The sleep measures in these studies varied considerably, with most studies using stable scales (11). A few of the studies used questionnaires produced by the authors themselves (4), and two studies used a combination of a maturity scale and a homemade questionnaire. Of these studies, 13 provided ORs for sleep disorders and suicidal ideation, 7 reported ORs for sleep disorders and suicide attempt, and only 2 studies showed the results of sleep disorders and completed suicide. Moreover, 17 studies reported patient characteristics and the risk between sleep disorders and suicidal behaviours for both male and female subjects, whereas one study only reported female subjects’ relevant outcomes. The characteristics of the included studies are shown in Table 1.

**Table 1:** Characteristics of included studies

Author	Country	Study design	Sample size	Diagnoses	Diagnostic criteria	Age (years)	Sleep disturbance	Sleep measure	Suicide outcome	Adjustments
Agargun et al. 1997 [28]	Turkey	Retrospective	113	Major depression	DSM-III-R	18~70 Mean: 32.6	Hypersomnia, insomnia	SADS	Suicidal ideation	NA
Agargun et al. 1997 [29]	Turkey	Prospective	41	Major depression	DSM-III-R	34.6 ± 10.8	Sleep quality, sleep latency, sleep duration, habitual sleep efficiency	PSQI	SADS suicidality score	NA
Li et al. 2012 [22]	Hong Kong	Prospective	419	Major depression	ICD-10	44.6 ± 10.4	Nightmares, insomnia	Sleep questionnaire, NDQ	Suicidal ideation	Yes
Lopes et al. 2016 [30]	Brazil	Cross-sectional	214	Major depression	DSM-IV	12.5 ± 3.1	Early awakening, night awakening, initial insomnia, daytime sleepiness	DICA-IV	Suicidal ideation, suicide attempts, suicide behavior, suicidal plan	Yes
Yoshimasu et al. 2006 [31]	Japan	Cross-sectional	231	Major depression	DSM-IV	36.3 ± 14.8	Insomnia, overall sleep disorders	Patients’ three most painful complaints, SDS, KMI	Suicidal ideation	Yes
Agargun et al. 1998 [32]	Turkey	Prospective	63	Major depression	DSM-III-R	34.1 ± 10.9	Nightmares	PSQI	Suicidal ideation	NA
Sit et al. 2015 [33]	United States	Retrospective	628	Depression	DSM-IV	28.7 ± 6.00	Sleep disturbance	SIGH-ADS	Suicidal ideation	Yes
Yoshimasu et al. 2006 [34]	Japan	Cross-sectional	199	Major depression	DSM-IV	38.4 ± 16.5	Sleep disorders	Patients’ chief complaints, KMI, SDS	Suicidal ideation	Yes
Agargun et al. 2007 [35]	Turkey	Retrospective	100	Major depression	DSM-IV	32.1 ± 10.7	Nightmares, insomnia	ICSD-R, HDRS	Suicide attempts	Yes
Stubbs et al. 2016 [36]	United Kingdom	Retrospective	5701	Depression	DSM-IV	43.4 ± 16.6	Sleep disturbance	Self-reported information	Suicidal ideation, suicide attempts	Yes
Gallagher et al. 2009 [23]	United Kingdom	Retrospective	163,512	Depression	DSM-IV	39.2 ± 18.0 (Female)	Insomnia	NA	Suicidal ideation, suicide attempts, completed suicide	Yes
Chellappa et al. 2007 [18]	Brazil	Cross-sectional	70	Major depression	DSM-IV	40.5 ± 12.5	Insomnia	SHQ, ICSD	Suicidal ideation	Yes
Park et al. 2016 [37]	Ten Asian countries/area	Retrospective	1122	Depression	ICD-10	48.1 ± 15.6	Insomnia	NIHCEG	Suicidal ideation	Yes
Guo et al. 2017 [38]	China	Cross-sectional	20,130	Depression	CES-D	16.3 ± 2.1	Sleep duration	Self-reported information	Suicide attempts, suicidal ideation	Yes
Nrugham et al. 2008 [39]	Norway	Prospective	2464	Depression	DSM-III-R	13.7 ± 0.5	Insomnia	KSADS-PL	Suicide attempts	Yes
McGirr et al. 2007 [40]	Canada	Prospective	156	Major depression	DSM-IV	42.4 ± 13.2	Insomnia, hypersomnia	Psychological autopsy method	Completed suicide	Yes
Eikelenboom et al. 2018 [10]	Netherlands	Prospective	1713	Major depression	DSM-IV	42.1 ± 12.3	Insomnia	IRS	Suicide attempts	Yes
Fang et al. 2019 [21]	China	Cross-sectional	2017	Major depression	DSM-IV	39.4 ± 12.5	Late insomnia, hypersomnia	A doctor-rating assessment questionnaire	Suicidal ideation	Yes

Ten Asian countries/area: China, Hong Kong, India, Indonesia, Japan, Korea, Malaysia, Singapore, Taiwan, and Thailand

*DSM* The diagnostic and statistical manual of mental disorders, *SADS* Schedule for affective disorders and schizophrenia, *HDRS* Hamilton depression rating scale, *PSQI* Pittsburgh sleep quality index, *ICD-10* International classification of diseases, tenth revision, *NDQ* Nightmare distress questionnaire, *DICA-IV* Diagnostic interview for children and adolescent DSM-IV version, *SDS* Self-rating depression scale, *KMI* Kyudai medical inventory, *SIGH-ADS* Structured interview guide for the Hamilton rating scale for depression, atypical depression symptoms, *ICSD-R* International classification of sleep disorders-revised, *SHQ* Sleep habits questionnaire, *NIHCEG* National Institute for Health and Clinical Excellence Guidelines, *CES-D* Center for epidemiology scale for depression, *IRS* Insomnia rating scale, *NA* Not available

### Evaluation of risk of bias

**Fig. 2:**
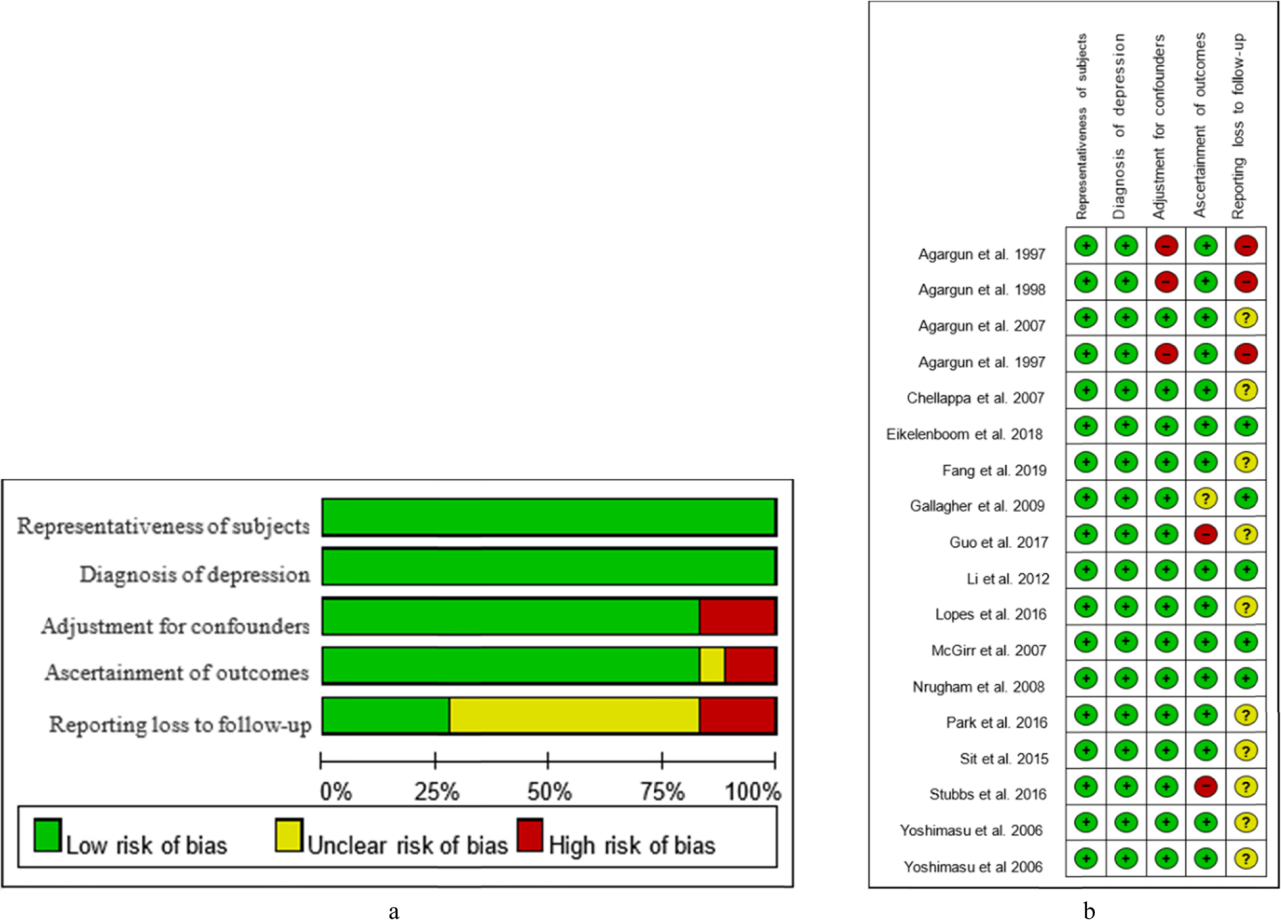
Assessment of the risk of bias. **a** Risk of bias graph and **b** Risk of bias summary; **a** represents the percentage of each bias level for five items, and **b** indicates the level of specific items in each study. Different colours (green, red, yellow) and symbols (“+”, “ - “, “?”) indicate “low risk of bias”, “high risk of bias” and “unclear risk of bias”. A study with three or more green “+” can be considered to have a low risk of bias

The risk of bias varied across the included studies. We found that most studies showed a low risk of bias in the representativeness of subjects (17) and the diagnosis of depression (18). Three studies did not control for confounding factors; thus, they were identified as “high risk” for this item. Fifteen studies were considered to have a low risk of bias in the ascertainment of outcomes due to their objective outcomes. However, only five studies reported follow-up and loss of follow-up, and similar information was not available in most studies (10). Overall, the included studies could be identified as having a low risk of bias because their NOS scores were 3 points or above. The details of the methodology for bias assessment in the overall study and for each study are shown in Fig. 2.

### Outcome and subgroup analysis

**Fig. 3:**
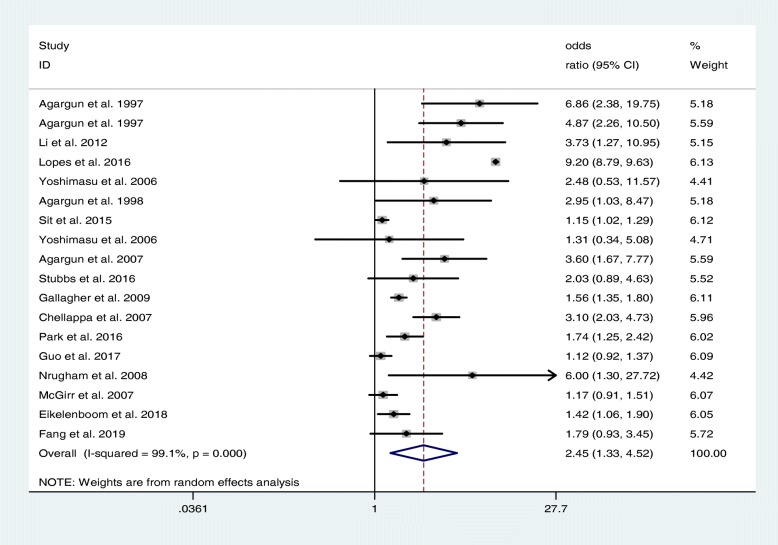
Forest plot of the relationship between sleep disorders and suicidal behaviour in depressed patients, *N* = 198,893. The data are expressed as odds ratios and the corresponding 95% confidence intervals. Pooled effect evaluation, represented by a diamond, was obtained with a random effects model. The heterogeneity between studies was explored by Cochran’s Q test and the I^2^ statistic, where I^2^ > 50% was considered evidence of substantial heterogeneity

After combining the ORs reported in the 18 included studies, we found that sleep disorders were associated with a significant risk of suicidal behaviour across all depressed patients in the random effects model. The pooled OR was 2.45 (95% CI: 1.33 4.52), and substantial heterogeneity was detected (I^2^ = 99.1%. *P* <  0.001) (Fig. 3). The meta-regression analysis showed that the R^2^ of the depression diagnostic outcome and suicidal behaviour type was 39.99% (*P* = 0.011) and 48.39% (*P* = 0.003), respectively. These variables were the cause of partial heterogeneity among studies; that is, a depression diagnosis could explain 39.99% of the heterogeneous sources, and the type of suicidal behaviour could explain 48.39% of the heterogeneous sources. The year of publication, study design, sample size, age of subjects, diagnostic criteria and sleep disorder type failed to explain the heterogeneity among studies (*P* > 0.05) (Additional file 1: Table S3).

In the subgroup analysis, we found that patients diagnosed with major depression (OR = 2.92, 95% CI:1.42 5.98) had a higher risk of suicidal behaviour than those diagnosed with depression (OR = 1.41, 95% CI:1.14 1.73). Studies with the DSM-III-R as diagnostic criteria (OR = 4.80, 95% CI: 2.90 7.67) showed a higher risk of suicidal behaviour, followed by those with the ICD-10 (OR = 2.13, 95% CI: 1.10 4.12) and the DSM-IV (OR = 2.11, 95% CI: 0.96 4.65). For the variable “sleep disorder type”, we found that insomnia (OR = 2.29, 95% CI:1.69 3.10) and nightmares (OR = 4.47, 95% CI:2.00 9.97) were significantly associated with an increased risk of suicidal behaviour. This relationship was not found for hypersomnia or other types of sleep disorders. Analysis based on the “suicidal behaviour type” subgroup showed that sleep disorders had a significant risk association with suicidal ideation (OR = 2.32, 95% CI:1.11 4.88) and suicide attempt (OR = 2.41, 95% CI:1.45 4.02). This association also existed with completed suicide but at the margin of error (*P* = 0.05). In addition, cross-sectional studies published between 1997 and 2010 and with a sample size < 1000 as well as with a mean age of patients < 40 detected a higher risk of suicidal behaviour. The details of the subgroup analysis are shown in Table 2.

**Table 2:** Subgroup meta-analysis and analysis of heterogeneity

Subgroup	No. of studies	No. of subjects	OR (95%CI)	Heterogeneity		*P* value
				I^2^(%)	*P* value	
Year of publication						
1997 ~ 2010	10	166,949	2.53 (1.75 3.66)	76.8	< 0.001	< 0.001
2010 ~ 2019	8	31,944	2.10 (0.80 5.53)	99.5	< 0.001	0.134
Study design						
Retrospective	6	171,176	1.79 (1.33 2.42)	83.1	< 0.001	< 0.001
Prospective	6	4856	2.25 (1.39 3.64)	75.3	0.001	0.001
Cross-sectional	6	22,861	2.45 (0.79 7.61)	98.9	< 0.001	0.122
Sample size						
< 1000	11	2234	2.99 (1.28 6.98)	99.2	< 0.001	0.011
> 1000	7	196,659	1.50 (1.24 1.81)	53.8	0.043	< 0.001
Age of subjects						
Mean < 40	12	189,712	2.75 (1.26 6.03)	99.4	< 0.001	0.011
Mean > 40	6	9181	1.81 (1.30 2.51)	73.4	0.002	< 0.001
Diagnostic criteria						
ICD-10	2	1541	2.13 (1.10 4.12)	43.2	0.185	0.025
DSM-III-R	4	2681	4.80 (2.90 7.67)	0	0.720	< 0.001
DSM-IV	11	174,541	2.11 (0.96 4.65)	99.4	< 0.001	0.063
CES-D	1	20,130	1.12 (0.92 1.37)	–	–	0.265
Diagnostic outcome						
Major depression	12	5336	2.92(1.42 5.98)	97.5	< 0.001	0.003
Depression	6	193,557	1.41(1.14 1.73)	75.6	0.001	0.001
Sleep disorder type						
Insomnia	12	172,759	2.29 (1.69 3.10)	86.1	< 0.001	< 0.001
Nightmares	3	582	4.47 (2.00 9.97)	0	0.490	< 0.001
Hypersomnia	3	2914	2.19 (0.77 6.21)	93.0	< 0.001	0.140
Other	7	27,144	2.41 (0.80 7.24)	99.6	< 0.001	0.117
Suicidal behavior type						
Suicidal ideation	13	194,419	2.32 (1.11 4.88)	99.2	< 0.001	0.026
Suicide attempts	7	193,834	2.41 (1.45 4.02)	87.7	< 0.001	0.001
Completed suicide	2	163,668	1.24 (1.00 1.53)	0	0.445	0.050
Other	2	225	4.19 (2.82 6.21)	0	0.654	< 0.001

*OR* Odds ratio, *ICD-10* International classification of diseases, tenth revision, *DSM* The diagnostic and statistical manual of mental disorders, *CES-D* Center for epidemiology scale for depression

### Publication bias and sensitivity analysis

There was visible asymmetry in the funnel plots, and Egger’s test was − 2.17 (*P* = 0.045). Combining the results of both analyses, we inferred that there was evidence of publication bias in this meta-analysis (Additional file 1: Figure S1).

Sensitivity analysis was performed after excluding five studies with a relatively high risk of bias. The pooled OR was 2.31 (95% CI: 1.12 4.77), which was slightly but not significantly lower than the previous pooled OR (OR = 2.45 95% CI: 1.33 4.52), indicating the stable and trustworthy nature of our analysis.

### GRADE assessment

The quality of the evidence was rated as very low for the association between sleep disorders and suicidal behaviour in the overall outcome and in the major depression subgroup. This evidence quality was increased by one point because the magnitude of the effect was large (OR > 2, based on consistent evidence from at least two studies with no plausible confounders). However, due to the substantial heterogeneity among studies and because publication bias was detected, which subtracted two points, the final score was one point, which represented a quality level of “very low”. Additionally, the evidence was assessed as low for the outcome of the depression subgroup. As with the status of evidence at the beginning of observational studies, no significant upgrade or downgrade factors were detected (Table 3).

**Table 3:** GRADE assessment of evidence quality

Quality assessment									Effect	Overall quality of evidence
No of studies	Study design	Risk of bias	Inconsistency	Indirectness	Imprecision	Publication bias	Other considerations	No of subjects	Relative (95% CI)	
Overall										
18	observational studies	not serious	Serious ^a^	not serious	not serious	detected	Very strong association ^b^	198,893	OR 2.45 (1.33 4.52)	⨁◯◯◯ VERY LOW
Major depression										
12	observational studies	not serious	Serious ^a^	not serious	not serious	detected	Very strong association ^b^	5336	OR 2.92 (1.42 5.98)	⨁◯◯◯ VERY LOW
Depression										
6	observational studies	not serious	not serious	not serious	not serious	not detected	none	193,557	OR 1.41 (1.14 1.73)	⨁⨁◯◯ LOW

*OR* Odds ratio, *CI* Confidence interval

^a^ The score was downgraded because substantial heterogeneity between studies was detected and could not be fully explained

^b^ The score was upgraded because the magnitude of the effect was large (OR > 2 based on consistent evidence from at least two studies, with no plausible confounders)

## Discussion

This work is the first meta-analysis to specifically assess the association between sleep disorders and suicidal behaviours in individuals with depression. Our evidence showed that patients with sleep disorders had a higher risk of suicidal behaviour than those without sleep disorders, and the more severe the depression was, the higher the risk of suicidal behaviour. Patients with sleep disorders were found to be more likely to exhibit various suicidal behaviours, including suicide ideation, suicide attempt and completed suicide. However, only insomnia and nightmares were found to be statistically associated with an increased risk of suicidal behaviour.

The causality between sleep disorders and suicidal behaviour in depression was not determined. Several hypotheses have been proposed in previous studies that might help explain the underlying mechanisms. Most researchers believe that the activity of 5-hydroxytryptamine (5-HT) is a major candidate for the cause of this association [29, 41]. 5-HT is an important neurotransmitter that promotes wakefulness and the onset of sleep by continuously inhibiting slow-wave sleep (SWS) and rapid eye movement sleep (REM), and its dysfunction might lead to sleep disorders [42]. Depressed patients were observed to have reduced SWS and a decreased concentration of 5-hydroxyindoleacetic acid (5-HIAA, a major metabolite of 5-HT) in cerebrospinal fluid. Benson et al. reported that the measurement of SWS in depression was closely related to serotonergic activity [43]. These results suggest that serotoninergic dysfunction might be a risk factor for sleep disorders. Furthermore, ritanserin, a specific 5-HT2 antagonist, has been confirmed to distinctly increase SWS in depression [44], which verifies the hypothesis that 5-HT plays an important role in sleep regulation. Moreover, a reduction in 5-HIAA has been found to indicate depression, modulate impulsive control and act as a risk marker for suicidal behaviour [45]. Therefore, serotonin dysfunction is presumed to be an important physiological factor for the association between sleep disorders and suicidal behaviour in depressed patients.

However, McCall et al. held that specific sleep disorders such as insomnia did not necessarily lead to suicide through serotonin. Instead, they proposed that sleep disorders destroy the function of serotonin. They found that insomnia symptoms appeared to be indirectly related to suicidal behaviours by dysfunctional beliefs and attitudes about sleep and nightmares [46]. In a clinical trial, Bernert et al. confirmed that less non-REM Stage 4 sleep and higher levels of nocturnal wakefulness were associated with suicidal behaviour in depressed patients [4]. In addition, other hypotheses, including the involvement of the hyperactive hypothalamic-pituitary-adrenal axis and the overactivity of the noradrenergic system, were also described as pathophysiological mechanisms of increased suicide risk in patients with depression and sleep disorders [18]. Because both of these hypotheses appear to be involved in the response to stressful events, causality is difficult to establish, and more evidence is needed to validate these hypotheses.

In addition to pathophysiological explanations, psychosocial factors such as unemployment, divorce and circadian rhythm disorders could directly aggravate sleep disorders in depression. However, the comorbidity among psychosocial factors, sleep disorders and suicidal behaviours remained an open question and needed to be addressed in a large number of cases [47]. Hopelessness might partly explain this relationship. Hopelessness is common in depression and is a key form of dysfunctional cognition that can lead to chronic insomnia and is itself a potential risk factor for suicide [9, 48]. Moreover, hopelessness can cause patients to have dysfunctional beliefs related to the hopelessness of sleep, which could aggravate the patient’s depression and suicidal tendencies. Other authors have hypothesized that sleep may provide an alternative to suicide for people with depression so that they can temporarily escape problems in their daily lives. Various sleep disorders may make these patients unable to escape and evade this burden except through suicidal behaviour [49]. There is evidence that loss of sleep might impair problem solving and mood regulation in patients and thus increase the risk of impulsive behaviour and suicidal tendencies [50].

In this meta-analysis, we found that the pooled OR of nightmares was almost twice that of insomnia, and both ORs were statistically significant. A body of work strongly suggests an association between insomnia and nightmares and suicidality in depression. A 4-year prospective observational study by Li et al. reported that in depressed outpatients who had been relieved of symptoms, the one-year prevalence of frequent insomnia at baseline and follow-up was 38.0 and 19.3%, respectively, and the corresponding incidence of nightmares was 24.0 and 9.3%, respectively. After controlling for age, marriage and psychiatric comorbidity, only nightmares were closely related to suicidal ideation [22]. Agargun et al. obtained a similar finding [35]. This result might be explained by a higher prevalence of and deeper psychological distress related to nightmares in suicidal patients.

Antidepressants, such as selective serotonin reuptake inhibitors (SSRIs) and serotonin-norepinephrine reuptake inhibitors (SRNIs), have been independently confirmed to be associated with nightmare recurrence after adjustment for confounding variables. Thus, depressed patients who take these medications are inevitably more likely to have nightmares [51]. Agargun et al. suggested that nightmares reflect a negative dream effect associated with deeper levels of self-criticism and self-blaming and make patients feel worse in the morning than later in the day and that the presence of terminal insomnia might play an important role in preventing morning depression [35].

Through an integrated motivational-volitional (IMV) model of suicidal behaviour, Russell et al. demonstrated that the relationship between insomnia and suicidal ideation was entirely governed by perceptions of defeat and entrapment. Nightmares were indirectly linked to suicidal ideation through perceptions of defeat and entrapment, but further research is required to untangle the link between insomnia/nightmares and defeat [52]. Overall, we did not find an independent predictive effect of hypersomnia on suicidal behaviour. Epidemiological data showed that respondents with simultaneous sleep disorders had a higher rate of suicide attempts than those with only hypersomnia [53]. Because daytime hypersomnia or fatigue might also be a symptom of depression, a careful exploration of hypersomnia in depressed patients is consistently recommended [48].

Another interesting outcome in this study was that depressed patients were at higher risk for the association between sleep disorders and suicide attempt, followed by the association between sleep disorders and suicidal ideation and completed suicide. This result is consistent with the findings of Malik et al. Remarkably, the pooled OR of each type of suicidal behaviour in our study was higher than that in their studies. This result might be related to interference from the many other mental illnesses they included, such as affective disorder, panic disorder, and post-traumatic stress disorder [54]. The process driving the difference between the three abovementioned suicidal behaviours and the risk of sleep disorders is unclear. As mentioned above, existing data suggest that nightmares, rather than other sleep disorders, have a stronger association with the risk of suicidal behaviour [55, 56]. In contrast, nightmares have been found to be more common among patients who have attempted suicide [35]. It might be suggested that nightmares could be considered an important indicator of suicide attempts in depressed patients [57]. In addition, sleep disorders were found to increase the risk of completed suicide, but the effect values were on the margins of statistical significance. Considering that few included studies could be combined, our results require further analysis after expanding the sample size.

Preventive interventions for suicidal behaviour have been poorly developed, especially for patients with depression or comorbidities with other mental illnesses [58]. Problem-solving therapy (PST) and cognitive behavioural therapy (CBT), as evidenced by a Cochrane Library review, effectively reduced the proportion of repeat self-injury, suicidal ideation and depressive symptoms within 12 months. However, the quality of this evidence was graded as moderate to low [59]. A study by Sayal et al. demonstrated that recruitment for a randomized controlled trial (RCT) of remotely delivered problem-solving CBT for young people with depression and repeated self-harm or suicide was not feasible because this population was unwilling to accept this intervention. They also found that clinician assessment following suicidal behaviour presentation mainly identified individuals with severe rather than mild-moderate depression [60].

In a study by Nadorff et al., the duration of nightmares was related to a higher risk of suicide. This finding might contribute to improving clinical practice [61]. In the past, clinicians were told not to use treatment related to nightmares, such as image rehearsal therapy, for people who were suicidal because the nightmares might worsen before they improve [62]. However, Nadorff et al. suggested that as long as appropriate precautions are taken (such as setting up a safety plan, hospitalization or frequent outpatient visits), it might be appropriate to use nightmare-related treatment for individuals at risk of suicide [63]. Moreover, some medications, such as clozapine and lithium, were confirmed to have anti-suicidal effects, and the latter had a beneficial effect on sleep. However, no mechanism was proposed for this drug to reduce suicidal behaviour by improving sleep [47, 58].

Although our outcomes showed significant differences (the ORs ranged from 1.24 to 2.41 for sleep disorders and 4.47 for nightmares), our results are still trustworthy due to the following factors. First, we established a rigorous search strategy to reduce any artificial omissions. Second, we adopted NOS to evaluate the methodological quality of each of the included studies and used the GRADE approach to evaluate the quality of the evidence. Third, most of the ORs we included were adjusted ORs, and through subgroup analysis and meta-regression, we found a partial source of heterogeneity among studies.

On the basis of statistical significance, this meta-analysis confirmed that the risk of suicidal behaviour across depressed patients was 2.45 times higher if they presented with sleep disorders than in patients without sleep disorders. This result is useful in clinical practice because in most areas, factors that increase the risk by a 10% threshold can be extremely important. However, regarding clinical significance, our findings need to be interpreted cautiously because of the low base rate of suicidal behaviour [63]. Our findings showed that patients with more severe depression have a higher risk of suicidal behaviour if they have sleep disorders. However, if the original study inadvertently included patients with bipolar disorder and these studies were mistakenly assigned to the “depression” group when the authors did not indicate that the subjects had major depression, this may have led to confusion in the outcomes of our two subgroups. In addition, previous studies confirmed that sleep disorders might be a symptom of major depression in patients [64, 65]. This makes it challenging for us to analyse sleep disorders and unipolar depression and may have led to an underestimation of the outcomes. Furthermore, considering that most of the participants in these included studies had unipolar depression, the discussion of this study focused on patients with unipolar depression and might be not applicable to studies involving other psychiatric disorders with depressive symptoms, such as bipolar disorder. In short, the present findings indicate a need for more powerful evidence. However, to clarify the conclusions, we must overcome the methodological limitations of existing articles to indicate whether the included participants have unipolar depression or bipolar disorder.

Furthermore, other limitations should be taken into account. First, all of the included studies were observational; thus, causality was difficult to establish. Second, there was still some heterogeneity that could not be explained, and the lack of uniform diagnostic criteria might reduce the comparability between studies. Third, because the data from many studies were not available, we did not have all the data from the 18 studies to summarize them. Thus, our results were an aggregation of potentially unstable estimates that were unlikely to be replicated. A more robust approach is needed to validate our results.

## Conclusions

This systematic review and meta-analysis distinctly indicated a link between sleep disorders and suicidal behaviour in depression. Insomnia and nightmares, in particular, were found to significantly increase the risk of suicide in depressed patients. Further research is needed to clarify the specific mechanisms of sleep disorders on suicidal behaviour, and more well-designed trials are needed to reduce the risk of suicide by improving sleep quality in depressed patients.

## Supplementary information

**Additional file 1: Table S1.** PRISMA Checklist. **Table S2.** Details of search strategy. **Table S3.** Meta-regression analysis. **Figure S1.** Funnel plots of the association between sleep disorders and suicidal behaviour. The solid line represents the overall pooled estimate for all included studies. Dashed lines represent 95% confidence intervals. Egger’s test gave a result of − 2.17, *P* = 0.045.

## Data Availability

The data sets supporting the conclusions of this article are included within the article and its supplementary materials.

## Abbreviations

CBT: Cognitive behavioural therapy
CES-D: Center for Epidemiology Scale for Depression
CI: Confidence interval
DSM: The Diagnostic and Statistical Manual of Mental Disorders
GRADE: Grading of Recommendations Assessment, Development and Evaluation
ICD-10: International Classification of Diseases, Tenth Revision
IMV: Integrated motivational-volitional
IOM: Institute of Medicine
MDD: Major depressive disorder
MeSH: Medical Subject Headings
NOS: Newcastle-Ottawa Scale
OR: Odds ratio
PRISMA: Systematic and Meta-analytical Preferred Reporting Program
PST: Problem-solving therapy
RCT: Randomized controlled trial
REM: Rapid eye movement sleep
RR: Relative risk
SRNI: Serotonin-norepinephrine reuptake inhibitors
SSRI: Selective serotonin reuptake inhibitors
SWS: Slow wave sleep
WHO: World Health Organization
