# Supplementary material for: Systematic review and meta-analysis of the relationship between sleep disorders and suicidal behaviour in patients with depression
Source: BMC Psychiatry. 2019 Oct 17;19:303. doi: 10.1186/s12888-019-2302-5 (PMC6798511; doi:10.1186/s12888-019-2302-5)
Supplement: Supplementary file 1 — Additional file 1: Table S1. PRISMA Checklist. Table S2. Details of search strategy. Table S3. Meta-regression analysis. Figure S1. Funnel plots of the association between sleep disorders and suicidal behaviour. The solid line represents the overall pooled estimate for all included studies. Dashed lines represent 95% confidence intervals. Egger’s test gave a result of − 2.17, P = 0.045. [file 12888_2019_2302_MOESM1_ESM.docx]

**Additional file 1**

Table S1. PRISMA Checklist

Table S2. Details of search strategy

Table S3. Meta-regression analysis

Figure S1. Funnel plots of the association between sleep disorder and suicide behavior

**Table S1. PRISMA Checklist**

| **Section/topic** | **#** | **Checklist item** | **Reported on page #** |
| --- | --- | --- | --- |
| **TITLE** | | | **1** |
| **Title** | **1** | **Identify the report as a systematic review, meta-analysis, or both.** | **1** |
| **ABSTRACT** | | | **1** |
| **Structured summary** | **2** | **Provide a structured summary including, as applicable: background; objectives; data sources; study eligibility criteria, participants, and interventions; study appraisal and synthesis methods; results; limitations; conclusions and implications of key findings; systematic review registration number.** | **1** |
| **INTRODUCTION** | | | **2** |
| **Rationale** | **3** | **Describe the rationale for the review in the context of what is already known.** | **2** |
| **Objectives** | **4** | **Provide an explicit statement of questions being addressed with reference to participants, interventions, comparisons, outcomes, and study design (PICOS).** | **3** |
| **METHODS** | | | **3** |
| **Protocol and registration** | **5** | **Indicate if a review protocol exists, if and where it can be accessed (e.g., Web address), and, if available, provide registration information including registration number.** | **4** |
| **Eligibility criteria** | **6** | **Specify study characteristics (e.g., PICOS, length of follow-up) and report characteristics (e.g., years considered, language, publication status) used as criteria for eligibility, giving rationale.** | **4** |
| **Information sources** | **7** | **Describe all information sources (e.g., databases with dates of coverage, contact with study authors to identify additional studies) in the search and date last searched.** | **4** |
| **Search** | **8** | **Present full electronic search strategy for at least one database, including any limits used, such that it could be repeated.** | **4** |
| **Study selection** | **9** | **State the process for selecting studies (i.e., screening, eligibility, included in systematic review, and, if applicable, included in the meta-analysis).** | **4** |
| **Data collection process** | **10** | **Describe method of data extraction from reports (e.g., piloted forms, independently, in duplicate) and any processes for obtaining and confirming data from investigators.** | **4** |
| **Data items** | **11** | **List and define all variables for which data were sought (e.g., PICOS, funding sources) and any assumptions and simplifications made.** | **5** |
| **Risk of bias in individual studies** | **12** | **Describe methods used for assessing risk of bias of individual studies (including specification of whether this was done at the study or outcome level), and how this information is to be used in any data synthesis.** | **5** |
| **Summary measures** | **13** | **State the principal summary measures (e.g., risk ratio, difference in means).** | **6** |
| **Synthesis of results** | **14** | **Describe the methods of handling data and combining results of studies, if done, including measures of consistency (e.g., I^2^) for each meta-analysis.** | **6** |

Page 1 of 2

| **Section/topic** | **#** | **Checklist item** | **Reported on page #** |
| --- | --- | --- | --- |
| **Risk of bias across studies** | **15** | **Specify any assessment of risk of bias that may affect the cumulative evidence (e.g., publication bias, selective reporting within studies).** | **6** |
| **Additional analyses** | **16** | **Describe methods of additional analyses (e.g., sensitivity or subgroup analyses, meta-regression), if done, indicating which were pre-specified.** | **7** |
| **RESULTS** | | | **7** |
| **Study selection** | **17** | **Give numbers of studies screened, assessed for eligibility, and included in the review, with reasons for exclusions at each stage, ideally with a flow diagram.** | **7** |
| **Study characteristics** | **18** | **For each study, present characteristics for which data were extracted (e.g., study size, PICOS, follow-up period) and provide the citations.** | **8** |
| **Risk of bias within studies** | **19** | **Present data on risk of bias of each study and, if available, any outcome level assessment (see item 12).** | **8** |
| **Results of individual studies** | **20** | **For all outcomes considered (benefits or harms), present, for each study: (a) simple summary data for each intervention group (b) effect estimates and confidence intervals, ideally with a forest plot.** | **8** |
| **Synthesis of results** | **21** | **Present results of each meta-analysis done, including confidence intervals and measures of consistency.** | **8** |
| **Risk of bias across studies** | **22** | **Present results of any assessment of risk of bias across studies (see Item 15).** | **8** |
| **Additional analysis** | **23** | **Give results of additional analyses, if done (e.g., sensitivity or subgroup analyses, meta-regression [see Item 16]).** | **9** |
| **DISCUSSION** | | | **10** |
| **Summary of evidence** | **24** | **Summarize the main findings including the strength of evidence for each main outcome; consider their relevance to key groups (e.g., healthcare providers, users, and policy makers).** | **10-14** |
| **Limitations** | **25** | **Discuss limitations at study and outcome level (e.g., risk of bias), and at review-level (e.g., incomplete retrieval of identified research, reporting bias).** | **15** |
| **Conclusions** | **26** | **Provide a general interpretation of the results in the context of other evidence, and implications for future research.** | **16** |
| **FUNDING** | | | **17** |
| **Funding** | **27** | **Describe sources of funding for the systematic review and other support (e.g., supply of data); role of funders for the systematic review.** | **17** |

***From:*  Moher D, Liberati A, Tetzlaff J, Altman DG, The PRISMA Group (2009). Preferred Reporting Items for Systematic Reviews and Meta-Analyses: The PRISMA Statement. PLoS Med 6(7): e1000097. doi:10.1371/journal.pmed1000097. For more information, visit:** [**www.prisma-statement.org**](http://www.prisma-statement.org)**.**

**Table S2. Details of search strategy**

| **Database** | **Search Period** | **Search Terms** |
| --- | --- | --- |
| **PubMed** | From inception to January 1, 2019 | ((((((((((((((((((((((((((Dyssomnias[MeSH Terms]) OR Dyssomnias[Title/Abstract]) OR Sleep Wake Disorders[MeSH Terms]) OR Sleep Wake Disorders[Title/Abstract]) OR (Sleep Initiation[Title/Abstract] OR Maintenance Disorders[Title/Abstract])) OR (Sleep Initiation and Maintenance Disorders[MeSH Terms])) OR insomnia[MeSH Terms]) OR insomnia[Title/Abstract]) OR Sleep arousal Disorders[MeSH Terms]) OR Sleep arousal Disorders[Title/Abstract]) OR Sleep Arousal Disorders[MeSH Terms]) OR Sleep Arousal Disorders[Title/Abstract]) OR Sleep Deprivation[MeSH Terms]) OR Sleep Deprivation[Title/Abstract]) OR Sleep Apnea[MeSH Terms]) OR Sleep Apnea[Title/Abstract]) OR poor sleep quality[Title/Abstract]) OR sleep disturbance[Title/Abstract]) OR sleeper syndrome[Title/Abstract]) OR sleep initiation dysfunction[Title/Abstract]) OR sleep dysfunction[Title/Abstract]) OR early awakening[Title/Abstract]) OR sleeplessness[Title/Abstract]) OR hypersomnia insomnia[Title/Abstract])) AND (Dyssomnias OR Sleep Wake Disorders OR Sleep Initiation "and" Maintenance Disorders OR insomnia OR Sleep arousal Disorders OR Sleep Disorders OR sleep deprivation OR Sleep Apnea OR poor sleep quality OR sleep disturbance OR sleeper syndrome OR sleep initiation dysfunction OR sleep dysfunction OR early awakening OR sleeplessness OR hypersomnia insomnia)) AND ((((((suicidal behavior[Title/Abstract]) OR Suicide[MeSH Terms]) OR Suicide[Title/Abstract]) OR Suicide Attempted[MeSH Terms]) OR Suicide Attempted[Title/Abstract]) OR suicide ideations[Title/Abstract]) |
| **Embase** | From inception to January 1, 2019 | **#**31. #17 AND #25 AND #30  #30. #26 OR #27 OR #28 OR #29  #29. 'suicide ideations':ab,ti  #28. 'suicide attempt':ab,ti  #27. 'suicide':ab,ti  #26. 'suicidal behavior':ab,ti  #25. #18 OR #19 OR #21 OR #22 OR #23 OR #24  #24. 'mental illness':ab,ti  #23. 'psychiatric disorders':ab,ti  #22. 'major depressive disorder':ab,ti  #21. 'tristimania':ab,ti  #20. 'mental disorders':ab,ti  #19. 'depression':ab,ti  #18. 'depressive disorder':ab,ti  #17. #1 OR #2 OR #3 OR #4 OR #5 OR #6 OR #7 OR #8 OR #9 OR #10 OR #11 OR #12 OR #13 OR #14 OR #15 OR #16  #16. 'sleep apnea':ab,ti  #15. 'sleep deprivation':ab,ti  #14. 'sleep disorders':ab,ti  #13. 'sleep arousal disorders':ab,ti  #12. 'insomnia':ab,ti  #11. 'sleep initiation and maintenance disorders':ab,ti  #10. 'sleep wake disorders':ab,ti  #9. 'dyssomnias':ab,ti  #8. 'hypersomnia insomnia':ab,ti  #7. 'sleeplessness':ab,ti  #6. 'early awakening':ab,ti  #5. 'sleep dysfunction':ab,ti  #4. 'sleep initiation dysfunction':ab,ti  #3. 'sleeper syndrome':ab,ti  #2. 'sleep disturbance':ab,ti  #1. 'poor sleep quality':ab,ti |
| **The Cochrane Library** | From inception to January 1, 2019 | #1 MeSH descriptor: [Depression] explode all trees  #2 (Depressive Disorder):ti,ab,kw OR (depression):ti,ab,kw OR (Mental Disorders):ti,ab,kw OR (tristimania):ti,ab,kw OR (Major Depressive Disorder):ti,ab,kw (Word variations have been searched)  #3 MeSH descriptor: [Suicide] explode all trees  #4 (suicidal behavior):ti,ab,kw OR (suicide ideations):ti,ab,kw OR (Suicide attempt):ti,ab,kw OR (Suicide):ti,ab,kw (Word variations have been searched)  #5 MeSH descriptor: [Sleep Wake Disorders] explode all trees  #6 MeSH descriptor: [Sleep Deprivation] explode all trees  #7 MeSH descriptor: [Sleep Apnea Syndromes] explode all trees  #8 MeSH descriptor: [Sleep Arousal Disorders] explode all trees  #9 (poor sleep quality):ti,ab,kw OR (sleep disturbance):ti,ab,kw OR (sleeper syndrome):ti,ab,kw OR (sleep initiation dysfunction):ti,ab,kw OR (sleep dysfunction):ti,ab,kw (Word variations have been searched)  #10 (Sleep Disorders):ti,ab,kw OR (sleep deprivation):ti,ab,kw OR (Sleep Apnea):ti,ab,kw OR (hypersomnia insomnia):ti,ab,kw OR (sleeplessness):ti,ab,kw (Word variations have been searched)  #11 (#1 OR #2)  #12 (#3 OR #4)  #13 (#5 OR #6 OR #7 OR #8 OR #9 OR #10)  #14 (#11 AND #12 AND #13) |

**Table S3. Meta-regression analysis**

| **Variable** | ***R^2^* (%)** | ***P value*** | **Exp(b)** | **Std. Err** | **t** | ***P value*** | **95%CI** |
| --- | --- | --- | --- | --- | --- | --- | --- |
| **Year of publication** |  |  |  |  |  |  |  |
| 1997 ~ 2010^*^ | -4.70 | 0.462 |  |  |  |  |  |
| 2010 ~ 2019 |  |  | 0.770 | 0.266 | -0.75 | 0.462 | 0.370 ~ 1.604 |
| **Study design** |  |  |  |  |  |  |  |
| Retrospective^*^ | -10.59 | 0.885 |  |  |  |  |  |
| Prospective |  |  | 1.165 | 0.505 | 0.35 | 0.729 | 0.462 ~ 2.936 |
| Cross-sectional |  |  | 1.223 | 0.521 | 0.47 | 0.642 | 0.494 ~ 3.033 |
| **Sample size** |  |  |  |  |  |  |  |
| < 1000^*^ | 14.75 | 0.100 |  |  |  |  |  |
| > 1000 |  |  | 0.570 | 0.184 | -1.74 | 0.100 | 0.288 ~ 1.129 |
| **Age of subjects** |  |  |  |  |  |  |  |
| Mean < 40^*^ | -1.30 | 0.374 |  |  |  |  |  |
| Mean > 40 |  |  | 0.724 | 0.256 | -0.91 | 0.374 | 0.342 ~ 1.532 |
| [**Diagnostic**](javascript:;) [**criteria**](javascript:;) |  |  |  |  |  |  |  |
| ICD-10^*^ | 7.64 | 0.226 |  |  |  |  |  |
| DSM-III-R |  |  | 2.101 | 1.344 | 1.16 | 0.265 | 0.533 ~ 8.287 |
| DSM-IV |  |  | 0.920 | 0.492 | -0.16 | 0.879 | 0.292 ~ 2.897 |
| CES-D |  |  | 0.486 | 0.380 | -0.92 | 0.372 | 0.904 ~ 2.607 |
| **Diagnostic outcome** |  |  |  |  |  |  |  |
| Major depressive disorder^*^ | 39.99 | 0.011 |  |  |  |  |  |
| Depression |  |  | 0.449 | 0.125 | -2.88 | 0.011 | 0.249 ~ 0.810 |
| **Sleep disturbance type** |  |  |  |  |  |  |  |
| Insomnia^*^ | 8.03 | 0.191 |  |  |  |  |  |
| Nightmares |  |  | 3.021 | 1.389 | 2.40 | 0.032 | 1.118 ~ 8.160 |
| Hypersomnia |  |  | 2.382 | 1.215 | 1.70 | 0.113 | 0.791 ~ 7.170 |
| Other |  |  | 2.695 | 1.314 | 2.03 | 0.063 | 0.940 ~ 7.728 |
| **Suicidal behaviors type** |  |  |  |  |  |  |  |
| Suicidal ideation^*^ | 48.39 | 0.003 |  |  |  |  |  |
| Suicide attempts |  |  | 1.953 | 0.610 | 2.14 | 0.051 | 0.995 ~ 3.833 |
| Completed suicide |  |  | 6.470 | 3.006 | 4.02 | 0.001 | 2.417 ~ 17.325 |
| Other |  |  | 3.576 | 1.425 | 3.20 | 0.007 | 1.511 ~ 8.459 |

*R^2^*: Adj R-squared, indicate the size of the heterogeneity between studies that can be explained by the covariates currently included in the model;

^*^: Control group; Std. Err: Standard error; CI: Confidence interval; ICD-10: International Classification of Diseases, Tenth Revision; DSM: The Diagnostic and Statistical Manual of Mental Disorders; CES-D: Center for Epidemiology Scale for Depression.

**Figure S1. Funnel plots of the association between sleep disorder and suicide behavior**

Figure S1. Funnel plots of the association between sleep disorder and suicide behavior
